# Platypnea-Orthodeoxia Syndrome and COVID-19 Successfully Treated With Percutaneous Patent Foramen Ovale Closure: A Report of Two Cases and Literature Review

**DOI:** 10.7759/cureus.56655

**Published:** 2024-03-21

**Authors:** Fernando Jiménez-Segovia, Sara Luis-García, Candela González-San Narciso, Pablo Demelo-Rodríguez, Rita García-Martínez, Francisco Galeano-Valle

**Affiliations:** 1 Internal Medicine, Hospital General Universitario Gregorio Marañón, Madrid, ESP

**Keywords:** sars-cov-2, platypnea-orthodeoxia syndrome, patent foramen ovale (pfo), intracardiac shunt, covid-19

## Abstract

Platypnea-orthodeoxia syndrome (POS) is a rare clinical condition characterized by positional dyspnea and/or hypoxia. We report two cases of patients with COVID-19 bronchopneumonia with a torpid evolution. Due to clinical suspicion of POS, a diagnostic workup was performed, including a bubble echocardiography, which revealed a patent foramen ovale (PFO) with early and massive passage of bubbles to the left cavities. Both patients underwent percutaneous PFO closure with a resolution of POS. Here, we present the second and third cases of POS associated with PFO successfully closed during the acute phase of COVID-19. This suggests that PFO closure could be a potential treatment option for this condition.

## Introduction

Platypnea-orthodeoxia syndrome (POS) is a rare clinical condition characterized by the appearance or worsening of hypoxemia (orthodeoxia) and dyspnea (platypnea) when moving from the supine position to the upright position of the trunk [1]. Hypoxemia is considered significant when there is a decrease in PaO2 of at least 4 mmHg or an oxygen desaturation of at least 5% upon transitioning from a supine position to sitting or standing [2].

The pathophysiology of POS is based on the mixing of venous blood with arterial blood through a connecting pathway or shunt, which can be intracardiac (e.g., patent foramen ovale (PFO)) or extracardiac (e.g., pulmonary arteriovenous fistula). For this to occur, therefore, a right-to-left shunt is necessary. In addition, a functional component that favors the R-L shunt is necessary when the patient passes from the decubitus position to the orthostatic position [2].

Since the beginning of the SARS-CoV-2 pandemic, a few cases of POS have been reported, and some authors have suggested that bronchopneumonia can cause an imbalance in ventilation/perfusion (V/P) that favors shunting in patients with PFO [3].

Here, we present two new cases of patients diagnosed with SARS-CoV-2 infection who subsequently developed POS due to a PFO. Additionally, we perform a narrative review of the literature on this topic.

## Case presentation

Case 1

A 74-year-old woman had a history of liver cirrhosis due to hepatitis B virus infection (without portal hypertension), intrinsic asthma, and mild pulmonary hypertension. She attended the emergency department in October 2020 due to fever, muscle pain, and dyspnea. Upon arrival, blood pressure was 122/78 mmHg, heart rate was 104 bpm, oxygen saturation was 88%, and temperature was 98.96ºF. The reverse transcriptase polymerase chain reaction (RT-PCR) for SARS-CoV-2 was positive. The chest X-ray showed bilateral bronchopneumonia. Blood analysis disclosed lymphocytes 0.5 103/μL (1.3-3.5 103/μL), fibrinogen 799 mg/dL (200-600 mg/dl), D-dimer 408 ng/mL (0-250 ng/ml), Nt-pro BNP 1017 ng/L (0-300 ng/L), C-reactive protein 18.5 mg/dL (<0.4 mg/dl), and ferritin 1094 μg/L (22-274 μg/L).

**Figure 1:**
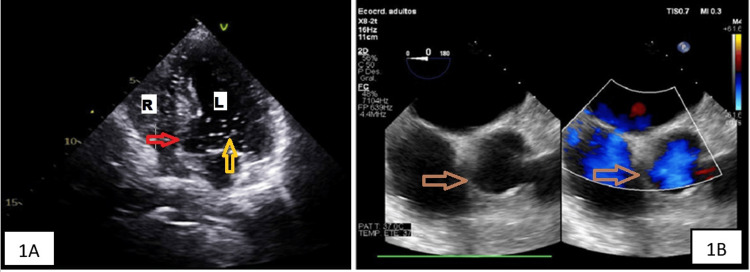
1A: Bubble echocardiogram demonstrating early and massive passage of bubbles from right to left cavities, suggesting intracavitary shunt compatible with the diagnosis of PFO in patient 1. 1B: PFO visible through transesophageal echocardiography R: right cavities, L: left cavities, PFO: patent foramen ovale, red arrow: PFO, yellow arrow: bubbles in left cavities, brown arrow: PFO visible through transesophageal echocardiography

**Figure 2:**
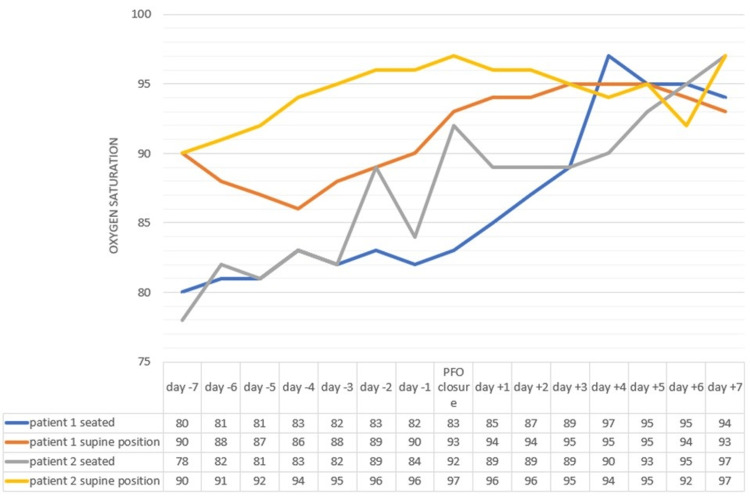
Oxygen saturation levels during hospitalization of patients 1 and 2 PFO: patent foramen ovale

The patient received treatment with dexamethasone and low-flow oxygen therapy. During the first two weeks of admission, the clinical condition improved slowly, but the chest X-ray only showed radiologically mild improvement. During the rehabilitation sessions, she presented episodes of oxygen desaturation (a decrease from 96% to 85%) and dyspnea when she was sitting, improving when she was in the supine position. A bubble contrast echocardiogram demonstrated the early and massive passage of bubbles to the left cavities, confirming the diagnosis of PFO (Figure 1A). In addition, it showed an atrial septum aneurysm with 1 cm of separation. A right heart catheterization showed a pulmonary capillary wedge pressure of 7 mmHg (4-12 mmHg) and a mean pulmonary artery pressure of 28 mmHg (<20 mmHg). Percutaneous closure was performed by means of an interatrial device with resolution of platypnea and orthodeoxia (Figure 2), allowing the patient to be discharged without oxygen therapy.

Case 2

A 61-year-old woman with no relevant personal history presented to the ED in March 2020 due to fever and dyspnea. Upon arrival, blood pressure was 103/69 mmHg, heart rate 112 bpm, oxygen saturation 84%, and temperature 102.2ºF. A nasopharyngeal swab RT-PCR for SARS-CoV-2 was positive. A chest X-ray showed bilateral bronchopneumonia, and blood analysis revealed: leukocytes 18.70 103/µL (4-10 103/µL), neutrophils 17.5 103/µL (1.8-7.5 103/µL), lymphocytes 0.9 103/µL (1.3-3.5 103/µL), INR 1.4 (0.8-1.2), fibrinogen >1000 mg/dL (200-600 mg/dl), D-dimer 654 ng/mL (0-250 ng/ml), procalcitonin 3.12 µg/L (0-0.5 µg/L), and C-reactive protein 59.3 mg/dL (<0.4 mg/dl).

The patient's respiratory condition worsened despite treatment with dexamethasone and high-flow oxygen therapy. A chest CT showed a bilateral subsegmental pulmonary embolism, and the patient was admitted to the critical care unit for invasive mechanical ventilation. After clinical stabilization (one month later), she was transferred back to the conventional hospital ward, where she developed typical symptoms of POS. During rehabilitation sessions, she presented episodes of oxygen desaturation and dyspnea that improved when she was in the supine position. A repeated chest CT showed signs of organized pneumonia, and a transesophageal echocardiogram revealed a PFO (Figure 1B). The PFO was successfully closed percutaneously, leading to clinical improvement in the patient (Figure 2).

## Discussion

We present two cases of POS in patients with a previously asymptomatic PFO in the setting of SARS-CoV-2 pneumonia, successfully treated with percutaneous PFO closure.

**Figure 3:**
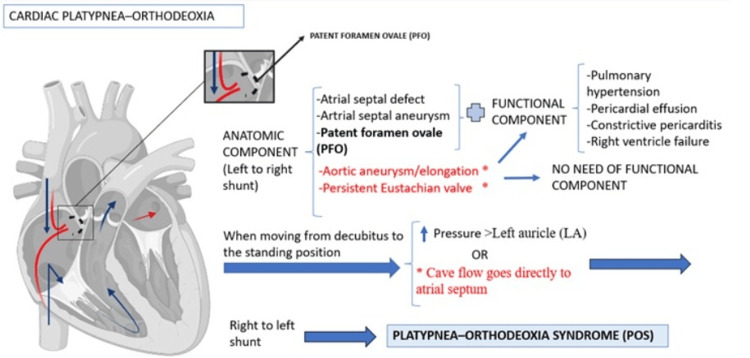
Mechanisms of cardiac platypnea-orthodeoxia Image Credit: Author

**Figure 4:**
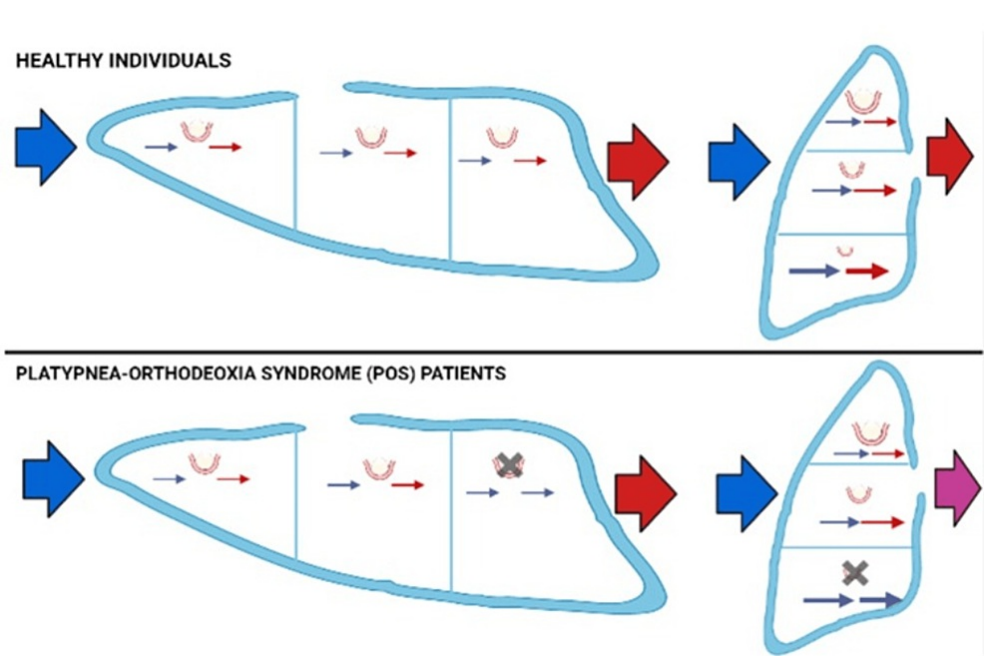
Mechanisms of pulmonary platypnea-orthodeoxia. V/Q and shunt in healthy individuals and in patients with POS Parenchymal lung diseases such as emphysema, interstitial lung disease, or consolidation with preferential involvement of lung bases can occasionally present as POS through severe V/Q mismatch, as discussed below. Pulmonary blood flow distribution is heterogeneous due to gravity. Gravity increases the blood flow in the lung bases more than in the apical regions, a phenomenon that is accentuated in upright positions. In addition, when standing upright, the right ventricular preload reduces, causing decreased output to the pulmonary arteries. This results in alveolar pressure exceeding pulmonary arterial and venous pressures at the lung apex. The lung apices tend to act as dead space in an upright position and contribute to an increase in V/Q mismatch and increased dyspnea, the so-called zone I phenomenon [5]. Lung diseases such as COVID-19 involve mostly the bases and posterior areas of the lung, causing intrapulmonary shunting and accentuating the V/Q mismatch. Blue arrow: deoxygenated blood, red arrow: oxygenated blood, purple arrow: mix of deoxygenated and oxygenated blood, black cross: interstitial disease, V/Q: ventilation/perfusion, POS: platypnea-orthodeoxia syndrome Image Credit: Author

The true incidence of POS is uncertain. POS requires two elements: on the one hand, an interatrial shunt (e.g.., PFO, an atrial septal defect, an atrial septal aneurysm, an aortic aneurysm/elongation, a persistent Eustachian valve) or an intrapulmonary shunt (e.g., hepatopulmonary syndrome in the context of cirrhosis, arteriovenous malformations in Rendu-Osler-Weber syndrome, or parenchimal lung diseases such as intersticial pneumonia, Pneumocystis jirovecii pneumonia, thoracic trauma, acute respiratory distress syndrome, pulmonary fibrosis, chronic obstructive pulmonary disease, and obstructive pneumonia such as bronchogenic or laryngeal carcinoma). On the other hand, a functional component is necessary that favors the R-L shunt when the patient changes position from supine to orthostatic. The functional component includes cardiac and pulmonary conditions. These conditions are proposed to cause transient elevations in right atrial pressures in the upright position. This reverses the pressure gradient across the intracardiac defect, which leads to an R-L shunt. Such a clinical scenario occurs in hypoxic lung diseases (e.g., pulmonary embolism), decreased right-sided compliance (e.g., right ventricular ischemia), or those associated with high right-sided filling pressures (e.g., myxoma of the right atrium or pericardial effusion) (Figure 3-4) [2,4].

The first patient had pulmonary conditions (cirrhosis, intrinsic asthma, mild pulmonary hypertension, and acute respiratory distress syndrome in the context of COVID-19 pneumonia), and the interatrial shunt was confirmed: a PFO associated with ascending aortic aneurysm (it is the most frequent coadjuvant anatomical alteration). Intracardiac POS can be seen in patients with aortic aneurysm, aortic root dilatation, and aortic elongation in patients with an atrial septal aneurysm [2,6]. The second patient had a pulmonary condition (acute respiratory distress syndrome in the context of COVID-19 pneumonia and the development of pulmonary embolism), and the interatrial shunt was a PFO as well.

The overall incidence of PFO was 27.3%, but it progressively declined with increasing age, from 34.3% during the first three decades of life to 25.4% during the fourth through eighth decades and to 20.2% during the ninth and 10th decades, with the vast majority being asymptomatic [7]. However, it can manifest as POS, or paradoxical embolism, in some cases. Its diagnosis requires an echocardiogram with agitated saline contrast showing an early passage of bubbles. The appearance of bubbles in the left atrium within three cardiac cycles suggests an intracardiac shunt [8]. In suspected POS cases, it should ideally be performed dynamically, first with the patient in a decubitus position and then repeated with the patient in a seated position. In cases of high suspicion with an indeterminate echocardiographic study, cardiac magnetic resonance imaging may be used to look for distortion of the cardiac anatomy leading to R-L shunting [9].

In patients with SARS-CoV-2 pneumonia, involvement of the lower and posterior zones of the pulmonary parenchyma and gravitational shunts of blood in the lower zones are frequent, hindering proper ventilation. This situation is aggravated by the presence of microthrombosis and microangiopathy observed in cases of severe infection [3], as was confirmed in the second patient. Patients with SARS-CoV-2 pneumonia present variable degrees of pulmonary fibrosis and respiratory dysfunction. Lung lesions caused by SARS-CoV-2 are often located in the subpleural area of the lower lobes of both lungs, as was the case in our first patient. Since gravity causes blood flow to be directed to the lung bases, the apical lung areas show increased ventilatory dead space. Thus, basal parenchymal involvement may worsen this physiological imbalance of ventilation and perfusion, precipitating POS [3]. Furthermore, although SARS-CoV-2 pneumonia predominantly affects the basal lung segments of both lungs, it is not clear why POS is only present in rare cases of SARS-CoV-2 pneumonia [3]. Although both of our patients had an anatomic component (PFO), the syndrome only manifested after SARS-CoV-2 pneumonia, suggesting that the infection may have played a role in the development of POS (Figures 3-4).

We conducted a literature search in January 2023 using PubMed and Scopus. We searched for articles using the terms "platypnea-orthodeoxia syndrome," "COVID-19," "SARS-COV-2," "patent foramen ovale," "persistent foramen ovale," and "intracardiac shunt" in English. Thirteen results were screened for eligibility, and among them, 11 articles were single case reports, while two articles were case series of two and five patients, respectively. These cases are summarized in Table 1.

**Table 1:** Characteristics of patients with platypnea-orthodeoxia syndrome and COVID-19 ICU: intensive care unit, IMV: invasive mechanical ventilation, NA: not available, PFO: patent foramen ovale, POS: platypnea-orthodeoxia syndrome, CPAP: continuous positive airway pressure, PSV: pressure support ventilation, R-L: right-to-left

Author (year) (ref)	N	Gender	Mean age	Admission to the ICU	Oxygen support needed	Chest image	Echocardiography	Presence of PFO	PFO closure	Pulmonary embolism	Hepatopulmonary syndrome	Moment of diagnosis of POS	Treatment of POS
Tan et al. (2020) [10]	5	5/5 (100%) males	65 (54-71)	5/5 (100%)	IMV 5/5 (100%)	Ground glass opacities and consolidation in the posterior lung segments and lower lobes 5/5 (100%)	NA	NA	NA	1/5 (20%)	-	During physiotherapy	Respiratory rehabilitation + supplemental oxygen. POS resolved over a median of 17 days
Tham et al. (2020) [11]	2	2/2 (100%) males	69 and 63	2/2 (100%)	IMV 1/1 (50%). High-flow nasal cannula 1/1 (50%)	Patchy air space opacities and ground-glass attenuation of both lungs	2/2 (100%) normal	0	0	0	1/2 (50%) possible liver cirrhosis	During the commencement of rehabilitation, 18 and 9 days, respectively, after admission to the ICU	Respiratory rehabilitation + supplemental oxygen. POS resolved after 65 and 22 days respectively from the day of detection
Singh et al. (2020) [12]	1	Male	66	1	IMV	Bilateral peripheral ground-glass opacities in both upper and lower zones	Normal	0	0	NA	-	During the commencement of rehabilitation	Physiotherapy and intermittent oxygen therapy via cannula nasal at 2 L O2. POS improved after 7 days of physiotherapy and the patient was discharged on room air after 15 days of hospitalization
Siddique et al. (2021) [13]	1	Male	45	0	High-flow nasal cannula	Bronchopneumonia + pneumomediastinum	Yes. R-L shunt	0	0	0	-	NA	Oxygen therapy. POS remained
Oldani et al. (2021) [14]	1	Male	80	0	CPAP High-flow nasal cannula	Bilateral peripheral ground-glass opacities in lower zones	NA	NA	NA	NA	NA	18 days after the onset of COVID-19	NA
Vanhomwegen et al. (2021) [15]	1	Male	55	0	Oxygen therapy and Boussignac continuous positive airway pressure	Bilateral peripheral ground glass opacities with crazy paving patterns	Yes. PFO	1	0	NA	-	Three weeks after hospital admission	Oxygen therapy. Two weeks after discharge, POS disappeared
Kramer et al. (2021) [16]	1	Male	73	1	IMV	Bilateral opacities.	Yes. PFO	1	1	0	-	During rehabilitation	PFO closure. No need for oxygen therapy when discharged
Hoshi et al. (2021) [3]	1	Woman	73	0	OxyMask^TM^ (Southmedic Inc., Barrie, ON, Canada) at 7 L/min	Bilateral and peripheral predominant consolidation and an air bronchogram	Normal	0	0	NA	-	During the commencement of rehabilitation	Respiratory rehabilitation. The patient was discharged under home oxygen therapy with 0.5 L/min via a nasal cannula 28 days after admission
Dodson et al. (2021) [17]	1	Male	85	0	High-flow nasal cannula	Consolidation and ground-glass opacities involving most of the left lung	Yes. PFO and atrial septum aneurysm	1	0	NA	-	28 days after the diagnosis of COVID-19.	Graded progression to standing and supplemental oxygen increases when upright. The patient was discharged and his oxygen requirement was resolved on approximately day 78
Zanoni et al. (2022) [18]	1	Woman	76	0	Oxygen therapy, CPAP	Bilateral ground-glass opacities. + pneumomediastinum and fibrosis	Normal	0	0	1	-	Approximately 1 month after the diagnosis of COVID-19	Oxygen therapy, diuretics, anticoagulant therapy, azithromycin. POS remained
Aprea et al. (2022) [19]	1	Woman	82	0	CPAP high-flow nasal cannula PSV	Moderate to severe interstitial lung involvement is greater at the lung bases	Normal	0	0	0	-	On the third day of hospital admission	Respiratory rehabilitation + supplemental oxygen. Discharge on day 18
Asami-Noyama et al. (2022) [20]	1	Male	83	0	High-flow nasal cannula	Bilateral ground-glass opacities	Normal	0	0	NA	-	1 month after the diagnosis of COVID-19	Gradual improvement in oxygenation. POS remained
Yowesgaran et al. (2022) [21]	1	Woman	63	0	NA	NA	Yes. PFA	1	1	0	-	1 year after the diagnosis of COVID-19	PFA closure. No need for oxygen therapy when discharged
Jiménez-Segovia et al. (2024) (current case report)	2	2/2 (100%) Women	74 and 61	1/2 (50%)	1/2 (50%) IMV. 1/2 (50%) low-flow oxygen therapy	Bilateral peripheral interstitial opacities compatible with bronchopneumonia	Yes. 2/2 (100%) PFA	2	2	1/2 (50%)	-	During the rehabilitation sessions	PFA closure. No need for oxygen therapy when discharged

The majority of POS cases associated with COVID-19 were described in men (14/18 patients) older than 60 years old [10-12,14,16,17,20]. This may be explained because the development of POS seems to be associated with severe COVID-19, which is more frequent in men and the elderly. Besides, most cases required admission to the ICU and invasive mechanical ventilation [10-12,16].

Most cases of POS were diagnosed during rehabilitation sessions when the patient was moved from the supine position to the upright position, as was the case with our patients. PFO was confirmed in four out of 12 patients who underwent echocardiography [15-17,21]. Almost all these cases were diagnosed during the acute phase of the SARS-CoV-2 infection, as was the case with our patients. However, in one case [21], the diagnosis of POS was made one year after the diagnosis of COVID-19 due to the persistence of dyspnea. It is also noteworthy that in one case [17], the patient presented an atrial septal aneurysm, as was seen in our first case.

On the other hand, when we analyzed the rate of pulmonary embolism, we found only two cases [10,18]. This number may be underestimated if we consider that microthrombosis is one of the mechanisms that could also play a role in the development of POS in COVID-19, as it occurred in our second patient. One of the previous cases [11] had cirrhosis, and a possible hepatopulmonary syndrome was also postulated as one of the mechanisms contributing to POS in COVID-19.

In the aforementioned articles, the main mechanism suggested for POS is a ventilation-perfusion mismatch within the lesions of COVID-19 pneumonia. This mismatch is exacerbated by other conditions mentioned above, such as pulmonary embolism or hepatopulmonary syndrome.

The treatment applied in previous cases of POS secondary to SARS-CoV-2 pneumonia was respiratory rehabilitation and oxygen therapy, showing a variable degree of efficacy but with good results in most cases [10-12,15-17,21]. However, in three of the aforementioned articles, POS remained [13,18,20]. In two of the four previous cases of PFO and COVID-19 [15,17] respiratory rehabilitation and supplemental oxygen were enough, but the closure of the PFO was not pursued. The ACC/AHA guidelines recommend ASD closure in patients with RA and right ventricular enlargement (Class I). Smaller ASDs (diameter <5 mm) with no evidence of right ventricular enlargement or pulmonary hypertension do not require closure unless they are associated with POS or paradoxical embolism (Class IIa) [22]. However, in the other two cases [16,21], the PFO closure was necessary since respiratory rehabilitation was not effective with good results. A 10-20% increase in upright oxygen saturation has been reported after the corrective surgery [23]. Nevertheless, only in one case [16] was the closure of the PFO successfully performed during the acute phase of COVID-19. Therefore, we present the second and third cases of POS linked to PFO, whose closure was also made successfully during the acute phase of COVID-19; this could be a possible treatment in those patients with PFO and COVID-19 who do not respond to conservative management.

## Conclusions

We present two rare cases of POS and COVID-19, successfully treated with percutaneous PFO closure. In COVID-19 patients who do not evolve favorably, the diagnosis of POS should be considered and PFO should be ruled out. In confirmed cases, PFO closure may be an effective treatment to improve the respiratory situation.
